# Finding Missing Interactions of the *Arabidopsis thaliana* Root Stem Cell Niche Gene Regulatory Network

**DOI:** 10.3389/fpls.2013.00110

**Published:** 2013-04-30

**Authors:** Eugenio Azpeitia, Nathan Weinstein, Mariana Benítez, Luis Mendoza, Elena R. Alvarez-Buylla

**Affiliations:** ^1^Laboratorio de Genética Molecular, Desarrollo y Evolución de Plantas, Instituto de Ecología, Universidad Nacional Autónoma de MéxicoCiudad Universitaria, México DF, México; ^2^C3, Centro de Ciencias de la Complejidad, Universidad Nacional Autónoma de MéxicoMéxico DF, México; ^3^Instituto de Investigaciones Biomédicas, Universidad Nacional Autónoma de MéxicoCd. Universitaria, México DF, México; ^4^Departamento de Ecología de la Biodiversidad, Instituto de Ecología, Universidad Nacional Autónoma de MéxicoCiudad Universitaria, México DF, México

**Keywords:** gene regulatory networks, Boolean models and functions, root stem cell niche, incomplete networks, predictive modeling, *Arabidopsis thaliana*

## Abstract

Over the last few decades, the *Arabidopsis thaliana* root stem cell niche (RSCN) has become a model system for the study of plant development and stem cell niche dynamics. Currently, many of the molecular mechanisms involved in RSCN maintenance and development have been described. A few years ago, we published a gene regulatory network (GRN) model integrating this information. This model suggested that there were missing components or interactions. Upon updating the model, the observed stable gene configurations of the RSCN could not be recovered, indicating that there are additional missing components or interactions in the model. In fact, due to the lack of experimental data, GRNs inferred from published data are usually incomplete. However, predicting the location and nature of the missing data is a not trivial task. Here, we propose a set of procedures for detecting and predicting missing interactions in Boolean networks. We used these procedures to predict putative missing interactions in the *A. thaliana* RSCN network model. Using our approach, we identified three necessary interactions to recover the reported gene activation configurations that have been experimentally uncovered for the different cell types within the RSCN: (1) a regulation of *PHABULOSA* to restrict its expression domain to the vascular cells, (2) a self-regulation of *WOX5*, possibly by an indirect mechanism through the auxin signaling pathway, and (3) a positive regulation of *JACKDAW* by MAGPIE. The procedures proposed here greatly reduce the number of possible Boolean functions that are biologically meaningful and experimentally testable and that do not contradict previous data. We believe that these procedures can be used on any Boolean network. However, because the procedures were designed for the specific case of the RSCN, formal demonstrations of the procedures should be shown in future efforts.

## Introduction

**Figure 1:**
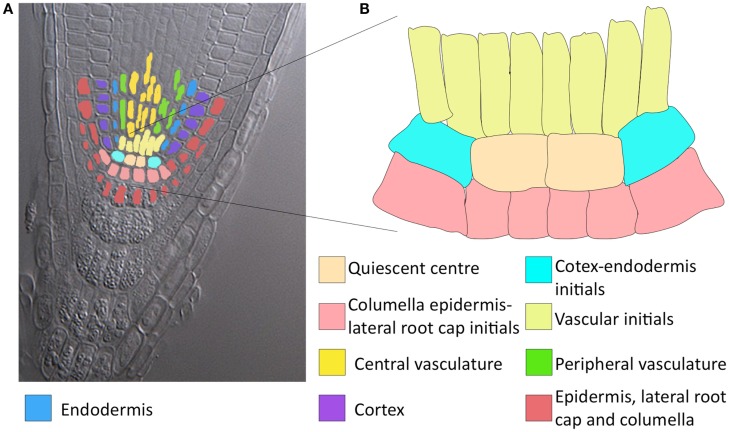
**Colour tracing of a confocal longitudinal section of an Arabidopsis root tip and a magnification of the RSCN**. **(A)** Cleared root tip of Arabidopsis thaliana. The expected stable gene configurations that characterise each cell type are distinguished with different colours. As observed, some of the expected attractors represent more than one cellular type. QC, quiescent centre; END, endodermis; VI, vascular initials; CEI, cortex-endodermis initials; COR, cortex; PVC, peripheral vascular cells; CLEI, collumela-epidermis-lateral-root-cap initials; LCC, collumela and lateral root cap; and CVC, central vascular cells. **(B)** Amplification of the RSCN.

The *Arabidopsis thaliana* root stem cell niche (RSCN) consists of a group of cells that rarely divide, known as the quiescent center, surrounded by four different types of stem cells (Figure 1; Dolan et al., 1993). The root stem cells produce all cell types necessary for the development of the primary root. Due to its architectural simplicity and its accessibility for experimental research at the genetic and molecular levels, the *A. thaliana* RSCN has become an important experimental model for molecular genetic studies in the last few decades. During this time, many important molecular mechanisms involved in the maintenance and development of the RSCN have been described (Sablowski, 2011; Azpeitia and Alvarez-Buylla, 2012). At least three molecular mechanisms have been uncovered as being fundamental for RSCN maintenance and development. The first mechanism involves auxin signaling and the PLETHORA (PLT) transcription factors that regulate auxin signaling (Galinha et al., 2007; Ding and Friml, 2010). The second mechanism involves the transcription factors SHORTROOT (SHR), SCARECROW (SCR), and some of their target genes (TGEN), as well as proteins that interact with them (Sabatini et al., 2003; Welch et al., 2007). The third mechanism includes CLAVATA-like 40 (CLE40) and WUSCHEL-RELATED HOMEOBOX 5 (WOX5; Stahl et al., 2009). Importantly, these three mechanisms are interconnected and present complex non-linear behaviors (reviewed in Azpeitia and Alvarez-Buylla, 2012).

Network models are an excellent tool for the integration and analysis of complex biomolecular systems, such as RSCN molecular mechanisms, at the structural and dynamic levels (de Jong, 2002; Alvarez-Buylla et al., 2007). In such models, the network nodes represent genes, proteins, RNA, or other molecular factors, while the edges correspond to positive or negative regulatory interactions among the nodes. Each node attains different values that correspond to its expression or activity level, and the node’s state changes in time depending on the state of the regulating nodes. The regulatory functions can be specified by different mathematical formalisms, but in all cases, these rules allow to follow the system’s collective dynamics over time and find relevant dynamic properties of the entire regulatory system. Among these properties, self-sustained network states, referred to as attractors, have been found to be particularly relevant. Attractors may be either cyclic or fixed-point.

Dynamic network models allow analyses of the sufficiency of reported data to explain the observed behaviors and properties of a particular system (de Jong, 2002). For example, Kauffman (1969) proposed that the attractors of a given gene regulatory network (GRN) could represent the experimentally observed gene expression profiles or configurations that characterize different cell types in biological systems. If the experimental data are sufficient, the GRN model attractors should coincide with the gene configurations experimentally documented for the different cell types. This hypothesis has been explored and validated with networks based on biological data (e.g., Mendoza and Alvarez-Buylla, 1998; Albert and Othmer, 2003; Espinosa-Soto et al., 2004). In fact, we published a GRN model of the RSCN a few years ago (Azpeitia et al., 2010).

Over the past few years, experimental reports have improved our knowledge about the RSCN GRN (reviewed in Azpeitia and Alvarez-Buylla, 2012). Interestingly, when we incorporated new experimental data, the set of attractors recovered by the model drastically changed. The new GRN model was not able to recover the observed attractors and generated many attractors that had not been observed experimentally. In this case, some key components or interactions are presumed to be missing. In principle, with the inclusion of putative missing components or interactions it should be possible to recover the expected attractors. However, the identification of the missing data in general is a non-trivial task.

In continuous systems, the inference of missing data is complicated partly because once the new information is introduced, new parameters must be estimated or incorporated into the postulated kinetic functions, and this procedure can cause the reformulation of such functions. In contrast, discrete networks usually do not need to deal with complicated parameter estimation or adjustment, and the redesign of the interaction functions is usually simpler. Boolean networks (BNs) are arguably the simplest discrete modeling approach for dynamic networks. In BNs, nodes may attain only one of two values or states: *0* if the node is OFF, and *1* if the node is ON. The level of expression for a given node may be represented by a discrete variable *x*, and its value at a particular time (*t* + τ) depends on the state of other components in the network (*x*_1_, *x*_2_, …, *x_n_*) at a previous time. The state of every node *x* therefore changes according to the following equation:

(1) $$x_{n}\;\left(t+\tau \right)=F_{n}\left(x_{n_{1}}\left(t\right),\;x_{n_{2}}\left(t\right),\;\ldots ,\;x_{n_{k}}\right)$$

**Figure 2:**
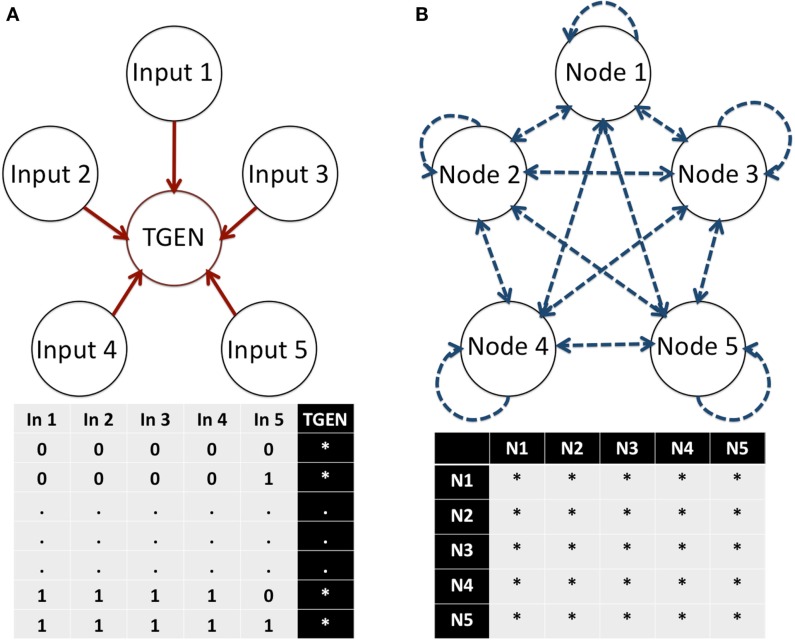
**Number of possible BFs in a node and the topologies of a network**. **(A)** The number of possible BFs for a particular node depends on the number of inputs or regulators of the node. In each possible state of its inputs, the node can assume a 0 or 1 expression value. Thus, $2^{2^{i}}$ possible BFs are available to describe the regulation of a node with i inputs. **(B)** The number of possible topologies of a network depends on the number of nodes. In a network, each node can interact with itself and any other node. Thus, *n*^2^ possible interactions exist. Because each interaction can either exist or not exist, $2^{n^{2}}$ possible topologies describe a network with N nodes. E, Exist, and D, Do not exist.

In this equation, $\left(x_{n_{1}}\left(t\right),x_{n_{2}}\left(t\right),\ldots ,x_{n_{k}}\left(t\right)\right)$ are the regulators of gene *x_n_*, and *F_n_* is a discrete function known as a Boolean function (BF). Such functions can be highly non-linear. Despite their simplicity, BN models have rich behaviors that yield meaningful information about the properties of the network under study. Because of this characteristic, BNs have been successfully used for the analysis of diverse GRNs (e.g., Albert and Othmer, 2003; Espinosa-Soto et al., 2004). The main constraint for the detection of putative missing interactions in BNs is that the number of possible BFs for a node increases as a double exponential function, namely $2^{2^{i}},$ where i represents the number of inputs regulating a target node. For example, a node with five regulatory inputs has 2^32^ (≈4 × 10^9^) possible BFs determining its dynamic response (Figure 2A). Similarly, the number of possible network topologies in a network is $2^{n^{2}},$ where n represents the number of nodes. Hence, in a BN with five nodes, a total of 2^25^ (≈3.35 × 10^7^) possible topologies determine the GRN connectivity (Figure 2B). Most GRN topologies can be described by different sets of BFs. Thus, if we consider a BN with five nodes where all nodes interact with each other in every possible manner, ${\left(2^{2^{i}}\right)}^{5}$ (≈1.46 × 10^48^) sets of BFs describe this topology. As observed, modeling the number of possibilities caused by additional components or links quickly becomes computationally intractable, even for such small networks using a simple Boolean formalism. Nevertheless, the dynamics of BNs with tens of nodes can be exhaustively analyzed in a relatively short amount of time, compared with other types of networks (e.g., Arellano et al., 2011). Thus, a methodology that allows for systematic integration and prediction of missing interactions in BNs would provide an instrumental tool in the proposal of a more complete RSCN GRN model and likely any other GRN.

Pal et al. (2005) studied how to produce a BN with a predefined set of expected attractors. Later, Zou (2010) studied how to obtain a set of expected attractors if the network topology exists and the BFs are partially known. Other researchers have investigated how to construct a BN from knowledge of the state-transition dynamics (e.g., Jarrah et al., 2007). Finally, Raeymaekers (2002) proposed that not all BFs are biologically meaningful and postulated a set of meaningful functions. The work of Raeymaekers is tightly linked to the so-called “canalizing BFs,” which produce stable and more biologically realistic BNs (Kauffman et al., 2004). Because the RSCN GRN model already relies on published experimental data, the methodology should be able to not only produce meaningful BFs, maintain the topology and recover the set of expected attractors but also agree with previous data regarding the reported molecular interactions. Moreover, taking into account reported molecular experimental data may greatly reduce the number of possible BFs to test. For example, SHR and SCR are known to directly and positively regulate *MAGPIE* (*MGP*) expression (Levesque et al., 2006; Cui et al., 2007); therefore, the BFs where SHR or SCR do not promote *MGP* expression directly do not need to be tested.

In this paper, we updated the RSCN GRN model using experimental data that were reported after we published our last model. Interestingly, when we incorporated the new experimental data, the set of recovered attractors did not correspond with the experimentally observed gene configuration states in the RSCN. Thus, we designed a set of procedures to add all possible missing interactions one-by-one to the model without contradicting experimental data and to greatly reduce the number of possible BFs when trying to predict missing interactions for a particular node. Using these procedures, we explored the effect of adding putative missing interactions in the set of attractors. We considered that the addition of a putative missing interaction improved our model if the interaction reduced the number of non-expected attractors or increased the number of expected attractors recovered by the model. The interaction that most improved the model, by removing non-expected attractors or adding expected attractors, was incorporated into the model. If more than one interaction equally improved the model, one interaction was randomly selected and added to the BN model. After the inclusion of an interaction, we repeated the process until the inclusion of three consecutive interactions did not improve the model, or we exclusively obtained the set of expected attractors. Based on our results, we proposed three putative missing interactions that were biologically meaningful, could be tested experimentally and in conjunction were sufficient to recover the set of observed attractors of the RSCN GRN; however, these interactions were not sufficient to eliminate the non-meaningful attractors in the model. Interestingly, these three interactions were always the first to appear as putative missing interactions. After adding the three interactions, the procedures produced more putative missing interactions that reduced the number of meaningless attractors. However, this second set of putative missing interactions was more variable, and we were never able to exclusively recover the set of expected attractors, strongly suggesting that additional components are yet to be discovered. Nevertheless, we provide three concise and testable predictions that are in agreement with the data that have been reported on RSCN patterning.

We believe that these procedures are useful for detecting missing interactions and possible incorrect gene regulatory or topological inferences due to incomplete data in any other GRN. However, because the procedures were generated *ad hoc* for the RSCN molecular interactions, generalization, and mathematical demonstrations of the procedures should be performed in the future to formally analyze the implications of using these procedures for any other GRN. Nevertheless, in the context of this study, we believe that these procedures may lead to novel research questions concerning general issues, such as the constraints that a given network topology imposes on the set of attractors.

## Methods

In this section, we describe the update to the RSCN GRN and the procedures used to reduce the number of possible BFs generated when trying to predict missing interactions and maintain previous experimental data. Then, we describe an evolutionary algorithm used to test the procedures in the GRN of the RSCN.

### RSCN GRN update

Three main regulatory mechanisms have been involved in the development and maintenance of the RSCN. The first mechanism involves the transcription factor SHR of the GRAS gene family (Sena et al., 2004). SHR is transcribed in the stele, but its protein moves to the adjacent cell layer (i.e., QC, cortex/endodermis initials, and endodermis) (Nakajima et al., 2001). In the QC, cortex/endodermis initials and endodermis, SHR promotes the expression of SCR, another GRAS transcription factor (Cui et al., 2007). SHR and SCR form a complex and together they regulate the expression of many genes, including other transcription factors and miRNAs. Their targets include the transcription factors *JKD* and *MGP*, as well as miRNA165/6 (Sozzani et al., 2010). JKD and MGP physically interact with SHR and SCR and are important for the regulation of *SCR* expression (Welch et al., 2007). The miRNA165/6 moves from its transcription domain and negatively regulates the expression of HD-ZIP III genes in the stele (Carlsbecker et al., 2010). The second mechanism is comprised of the auxin signaling pathway and their TGENs, such as the PLT transcription factors (Galinha et al., 2007). In the auxin signaling pathway, the transcription factors AUXIN RESPONSE FACTORS (ARF) form dimers with proteins of the Aux/IAA family (Guilfoyle and Hagen, 2007). In an Aux/IAA-ARF dimer, the ARFs cannot promote the expression of their TGENs. However, auxin promotes Aux/IAA degradation (Calderón Villalobos et al., 2012). Thus, as auxin concentration increases, the ARFs are released from the Aux/IAA negative regulation and promote the expression of their TGENs. The third mechanism involves the transcription factor WOX5, the mobile protein CLE40 (a negative regulator of WOX5) and their receptor ACR4 (Stahl et al., 2009, 2013). Importantly, these mechanisms interact with each other (Azpeitia et al., 2010).

**Figure 3:**
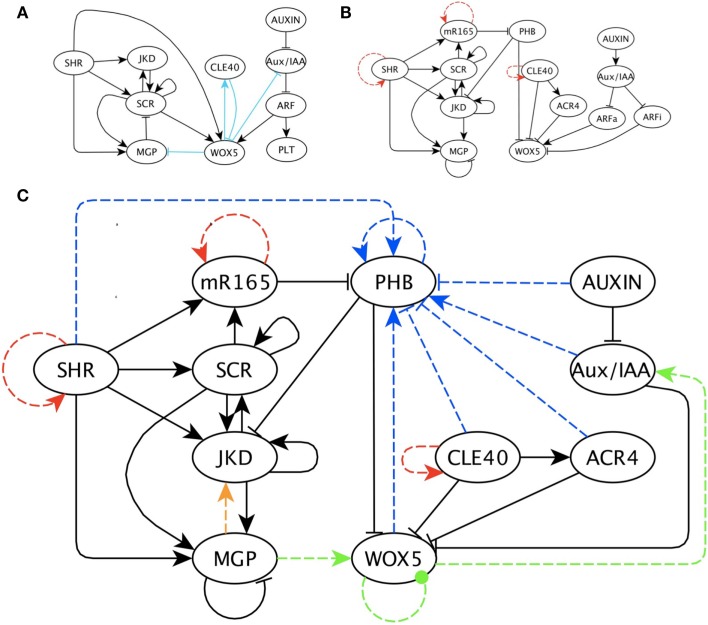
**The previous and updated RSCN GRN with predicted missing interactions**. **(A)** Previously published RSCN GRN (Azpeitia et al., 2010). The light blue edges indicate previous predicted missing interactions. **(B)** Updated RSCN GRN as explained in the main text. The red edges are the self-regulations introduced to represent protein movement. **(C)** RSCN GRN with predicted missing interactions. For simplicity and clarity, intermediary nodes were not included in this GRN; however, these nodes are available in Supplementary Material. Yellow, green and blue edges are the three predicted interactions required to recover the expected attractors and are grouped according to the node’s functions. The blue edges always indicate regulation of PHB. The yellow edge is a positive regulation of JKD by MGP. The green edges correspond to regulation of WOX5. The dotted green edge indicates a negative or positive regulation of WOX5 by itself.

To update our previous GRN, we first omitted the interactions predicted by our previous work that had not yet been confirmed experimentally and that were rather hypothetical (Azpeitia et al., 2010). The reason for this omission is that the objective of this work was to detect and predict missing interactions using a systematic approach that could be applied to any system. The only prediction in our previous model that we conserved is the negative regulation of WOX5 by CLE40 because this result was experimentally documented while the model was under review (Stahl et al., 2009). Then, we removed *PLT* genes from the model because even though these genes are essential for RSCN maintenance (Galinha et al., 2007), the *PLT* genes do not regulate any other node in the model under analysis and can therefore be collapsed (Figures 3A,B). We also corrected or completed data about the interactions among SCR, MGP, and JKD according to the results of Ogasawara et al. (2011). Thus, in this model, MGP does not act as a negative regulator of *SCR*; JKD is a positive regulator of *MGP* and itself; and MGP negatively self regulates (Ogasawara et al., 2011). Stahl et al. (2009) reported that the receptor ACR4 is necessary for CLE40 negative regulation of *WOX5* and is positively regulated by CLE40. Apparently, SHR and SCR regulation of *WOX5* is not direct (Sozzani et al., 2010). Moreover, SHR and SCR promote *miRNA165/6* expression, while miRNA165/6 represses PHB mRNA translation (Carlsbecker et al., 2010). According to Grigg et al. (2009), PHB overexpression prevents *WOX5* expression. Hence, we decided to delete the positive, direct regulation of *WOX5* by SHR and SCR because the regulation is not direct, and incorporate this positive regulation indirectly by the repression of PHB. Recently, PHB was reported to be a negative regulator of *JKD* (Miyashima et al., 2011). Because our model does not incorporate space explicitly, we replaced molecular diffusion and movement by including a positive self-regulatory edge in nodes that move among cells (i.e., SHR, CLE, and miRNA165/6) to allow expression of these nodes where they move and no node positively regulates them. Finally, we reduced the auxin signaling pathway to the auxin and Aux/IAA nodes because the pathway is composed of linear path-like interactions that can be collapsed. In this way, we reduced the number of nodes in our network, and this change reduced the number of possible topologies and BFs describing the network once we incorporated putative missing interactions. In Section “Appendix 1 in the Appendix” we present the data and results of the analysis performed to reduce the auxin signaling pathway. We incorporated this information in the updated regulatory network model proposed here (Figure 3B). The main experimental data about gene interactions are presented in Table 1.

**Table 1:** **Main experimental information used in the RSCN GRN**.

**INTERACTIONS**	**EXPERIMENTAL EVIDENCE**	**REFERENCE**
*SHR* → *SCR*	The expression of *SCR* is reduced in *shr* mutants background. ChIP-QRTPCR experiments show that *SHR* interacts *in vivo* with the predicted regulatory sequences of *SCR* and positively regulate it	Helariutta et al. (2000), Levesque et al. (2006), Cui et al. (2007)
*SCR* → *SCR*	In *scr* mutant background promoter activity of *SCR* is absent in the QC and CEI. A ChIP-PCR assay confirmed that *SCR* binds to its own promoter and promotes its own expression	Sabatini et al. (2003), Cui et al. (2007)
*JKD* → SCR	The SCR promoter expression in QC and CEI is not detected in JKD mutants from early heart stage onward. JKD was able to activate luciferase gene expression driven by a 1.5 kb SCR promoter region	Welch et al. (2007), Ogasawara et al. (2011)
*JKD* → JKD	JKD was able to activate luciferase gene expression driven by a 3.5 kb JKD promoter region	Ogasawara et al. (2011)
*JKD* → MGP	JKD was able to activate luciferase gene expression driven by a 3.5 kb MGP promoter region	Ogasawara et al. (2011)
*MGP* -| MGP	MGP addition was able to inhibit the SHR, SCR, and JKD induced luciferase gene expression driven by a 3.5 kb MGP promoter region	Ogasawara et al. (2011)
*SHR* → KD	The post-embryonic expression of JKD is reduced in shr roots. A CHIP-chip analysis detected JKD as a direct target gene of SHR	Welch et al. (2007), Cui et al. (2011)
*SCR* → JKD	The post-embryionic expression of JKD is reduced in scr roots	Welch et al. (2007)
*SCR* → *WOX5*	WOX5 expression is reduced in shr mutants	Sarkar et al. (2007)
*SHR* → *WOX5*	WOX5 expression is undetectable in scr mutants	Sarkar et al. (2007)
*Auxin signalin pathway* → WOX5	In mp or bdl mutants background WOX5 expression is rarely detected	Sarkar et al. (2007)
*Auxin signalin pathway* -| *WOX5*	In *iaa17* mutants background *WOX5* expression is decreased	Ding and Friml (2010)
*SCR* → *miRNA165/6*	In *scr* single mutants, miRNA165/6 expression is greatly reduced. A ChIP-PCR assay confirmed that *SCR* binds to miRNA165/6 promoter	Carlsbecker et al. (2010), Miyashima et al. (2011)
*SHR* → *miRNA165/6*	In *shr* single mutants, miRNA165/6 expression is greatly reduced. A ChIP-PCR assay confirmed that *SHR* binds to miRNA165/6 promoter	Carlsbecker et al. (2010), Miyashima et al. (2011)
*miRNA165/6* -| *PHB*	Over expression of *miRNA165/6* causes a decrease in the transcript levels of *PHB*. The allele *phb-1d* that expresses miRNA165/6-resistant PHB transcripts has ectopic *PHB* transcripts expression	Zhou et al. (2007), Miyashima et al. (2011)
*PHB* -| WOX5	In se mutants, which fail to repress PHB expression, embryonic WOX5 expression is absent	Grigg et al. (2009)
*PHB* → JKD	jkd transcripts levels are reduced in the phb-1d miRNA-resistant PHB allele	Miyashima et al. (2011)
*CLE40* → *ACR*	CLE40p treatment strongly increased *ACR* expression	Stahl et al. (2009)
*CLE40* -| *WOX5*	In *cle40* mutants the WOX5 expression domain is expanded, and CLE40p treatment reduced WOX5 expression in the QC	Stahl et al. (2009)
*SHR* → MGP	The expression of MGP is severely reduced in the shr background. Experimental data using various approaches have suggested that MGP is a direct target of SHR. This result was later confirmed by ChIP-PCR	Levesque et al. (2006), Cui et al. (2007, 2011), Welch et al. (2007)
*SCR* → *MGP*	SCR directly binds to the MGP promoter, and MGP expression is reduced in the scr mutant background	Levesque et al. (2006), Welch et al. (2007)

Importantly, the inclusion of a putative missing interaction in a node with four inputs was excessively demanding. To allow the addition of putative missing interactions in nodes with four inputs, we created intermediary nodes that integrate the influence of two regulators over any gene with four regulators (see Supplementary Material).

### Integrating and formalizing experimental data into BN models

As mentioned above, experimental data are formalized as BFs in BNs. BFs follow the equation:

$$x_{n}\left(t+\tau \right)=F_{n}\left(x_{n_{1}}\left(t\right),x_{n_{2}}\left(t\right),\ldots ,x_{n_{k}}\left(t\right)\right)$$

where *x_n_*(*t* + τ) represents the state of node *n* at time *t* + τ (τ representing a positive integer), and $\left(x_{n_{1}}\left(t\right),x_{n_{2}}\left(t\right),...,x_{n_{k}}\left(t\right)\right)$ represents the state of the regulators of node *x*_n_ at time *t*. BFs can be described either as logical statements or as truth tables. Logical statements use the logical operators AND, OR, and NOT, while the state of node *n* at time *t* + τ is given for all possible combinations of its *k* regulator states of activation at time *t* in truth tables. Using the BFs of all nodes, we can follow the dynamics of the GRN until it reaches a stationary network configuration or state (attractor). A network configuration is the vector comprised of a set of values, where each value corresponds to the state of a specific node of the network. Single-state, stationary configurations are known as fixed-point attractors, while a set of network states that orderly repeat among each other cyclically correspond to cyclic attractors. Importantly, fixed-point attractors usually correspond to the arrays of gene activation states that characterize different cell types. Once we recover the set of attractors in the GRN, we can compare the attractors with the expected attractors, which are the experimentally observed stable gene expression configurations. The expected set of attractors are defined from gene expression patterns obtained from the literature that clearly define the spatio-temporal gene configuration of the system. Different data types, such as that obtained from transcriptional and translational reporter assays and microarrays, can be used to define the expected attractors. If the experimental information is correct, but the recovered and the expected attractors are not the same, then the GRN is likely missing information. One possibility is that there are missing interactions within the network.

To add putative missing interactions, two important issues must be considered.

(1) One needs to understand how the experimental data are contained in the BFs. In general, more than one logical statement exists for most BFs. Importantly, such equivalent logical statements can use different logical operators. For example, the logical statement “RGEN1 AND RGEN2” that uses the AND operator is equivalent to the logical statement “NOT (NOT RGEN1 OR NOT RGEN2)” which uses the OR and NOT logical operators. In contrast, a unique truth table represents each BF, indicating that the truth table is not arbitrarily selected. Indeed, each logical statement has an equivalent representation in a truth table, while each truth table can have many equivalent logical statements. Thus, in this paper, we use truth tables to analyze how the experimental information is formalized and contained in the BFs.
(2) One needs to realize that the same BF can formalize regulatory interactions documented with various types of experimental data. For example, we can infer that TGEN is regulated by Regulatory Gene 1 (RGEN1) through a loss-of-function mutant analysis or with a chromatin immunoprecipitation assay. Consequently, we may need to preserve the information gathered from different experiments and then formalize the same BF. Thus, the procedure through which we add putative missing interactions while maintaining congruence with the available experimental data depends on the specific set of experimental data available. In this work, we generated four different procedures by analyzing how the experimental information of the RSCN GRN is contained in the truth tables. The procedures were designed as follows.

#### Procedure 1

Add a putative missing interaction generated by gain and loss-of-function mutants (Table 2). When this procedure is used, each row of the truth table represents an experiment, and we can only state that under certain conditions the TGEN responds differentially to changes in the expression levels of other genes.

**Table 2:** **Summary of the procedures proposed to infer putative missing interactions in data-based network models**.

Procedure	Application to inferring putative interactions
**PROCEDURE 1**	
Adding missing links in congruence with available experimental data that can be represented in single rows of truth tables	This is probably the most simple procedure. It allows modifying the network adding missing putative interactions, and at the same time the regulatory effects of the nodes whose role is based on experimental that is represented by single rows of the true tables is preserved. Examples of experiments represented by single rows are loss and gain-of-function mutants
**PROCEDURE 2**	
Adding missing links while maintaining the sign of the regulation	Prevents changes in the regulatory sign of genes when we introduce putative missing interactions
**PROCEDURE 3**	
Adding missing links while maintaining documented protein–protein interactions	This procedure guarantee that the joint action of proteins acting as complexes is respected in the new rows that result from the addition of new interactions. However, it allows new complexes to be formed, replacing, deleting or including one or more components in the complex
**PROCEDURE 4**	
Adding missing links while maintaining necessary protein–protein interactions	Procedure 4 is similar to procedure 3, since it also guarantee the joint action of proteins acting as complexes. However, this procedure do not allows the substitution or deletion of any of the components of the complexes. Importantly, it does allow the incorporation of other components in the complex
**PROCEDURE 5**	
Adding missing links without independent TGEN activity	Procedure 5 prevents the generation of BFs where one or more regulator has no effect on its target gene
**PROCEDURE 6**	
Adding missing links without ambiguous regulators	Procedure 6 prevents the emergence of nodes that act as global positive and negative (ambiguous) regulators at the same time

#### Procedure 2

Add a putative missing interaction to a truth table while maintaining the sign of the regulation of previously reported regulators (Table 2). Some experimental data clearly determine whether a gene is a positive or a negative regulator. When this case is true, we want to maintain that regulation with the same sign. Using this procedure, when we add a putative missing interaction, we exclusively generate BFs without changing the sign of the regulation of the RGENs that we want to maintain as positive or negative regulators.

#### Procedure 3

Add a putative missing interaction to a truth table while maintaining documented protein–protein interactions (Table 2). The experimental data can indicate that a pair or a group of genes act as complexes. However, this fact does not mean that all the proteins in a complex only function in the context of the complex. The proteins could act as individual units or form complexes with different proteins. This procedure allows putative missing interactions to be added while maintaining the functionality of the documented complexes. However, in the new BFs, the proteins in the complex can have new functionalities by themselves or with the putative missing regulator; the proteins can be substituted in or deleted from the complex under certain conditions; and new regulators can become part of the complex. For example, imagine a complex formed by proteins A and B. Once protein C is added as a putative missing regulator, the original protein A-B complex will continue to be a protein A-B complex, but now proteins A and B could also function in a protein A-C, B-C, or A-B-C complex.

#### Procedure 4

Add a putative missing interaction to a truth table where one or more of the nodes can act exclusively as part of a protein complex (Table 2). Contrary to the last procedure, the experimental data can indicate that a pair or a group of proteins are only functional when they work together. Using this procedure, we can maintain proteins as functional only when they form a complex, once a putative missing interaction is added. Importantly, proteins cannot be substituted or deleted from the complex under any condition. In contrast to procedure 3, the A-B complex cannot become an A-C or B-C complex. However, protein C could be included in the complex and function in an A-B-C complex.

We also designed two procedures that stem from the limits of the Boolean formalism, and we propose these procedures to simplify the interpretation of the predicted missing interactions. These procedures were designed as follows.

#### Procedure 5

Add a putative missing interaction while avoiding the generation of BFs where one or more nodes do not influence the activity of the target node (Raeymaekers, 2002) (Table 2). Notably, certain types of regulatory interactions cannot be expressed with a Boolean formalism (e.g., the modulation of protein activity by another protein). Thus, a TGEN may be regulated by a given RGEN even if this regulation is not explicitly reflected in the BFs. Given this uncertainty, we avoided generating these BFs.

#### Procedure 6

Add a putative missing interaction while avoiding the generation of BFs where one or more nodes act as positive and negative regulators in the same truth table (Raeymaekers, 2002) (Table 2). This assumption is a simplification because these types of regulatory interactions have been reported experimentally. However, these interactions appear to be infrequent, and exclusion of these interactions allowed us to greatly reduce the number of BFs when we added a putative missing interaction.

A more detailed description of the procedures and their design is available in Section “Appendix 2 in the Appendix.”

### Application of the procedures to postulate a set of possible new BFs given putative missing interactions in the *A. thaliana* RSCN GRN

To detect and predict missing interactions, we applied an evolutionary algorithm using the following steps.

**Figure 4:**
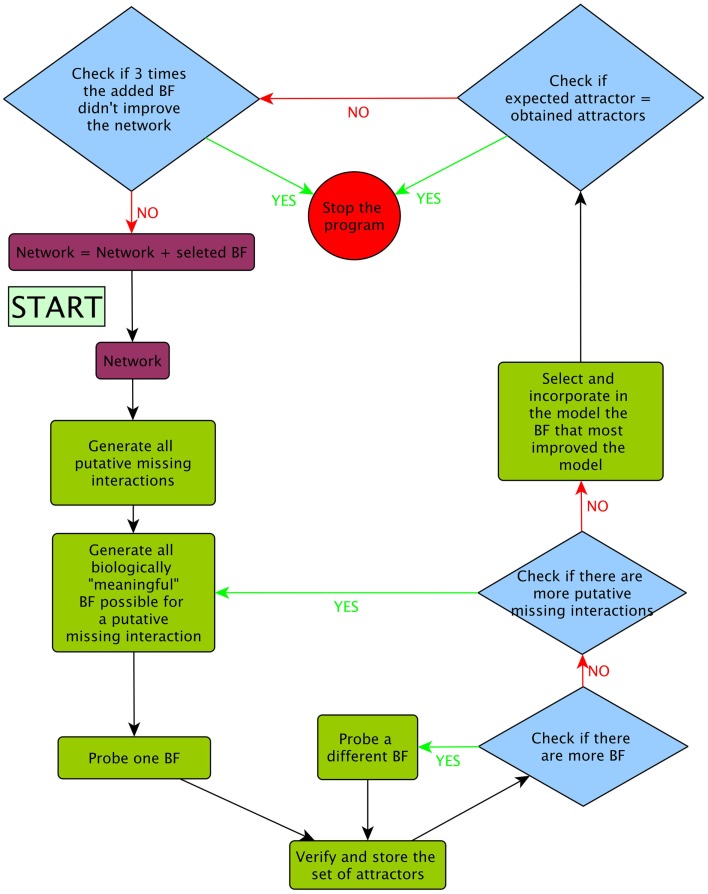
**Flux diagram of the evolutionary algorithm followed during the prediction of putative missing interactions using our procedures**.

**Figure 5:**
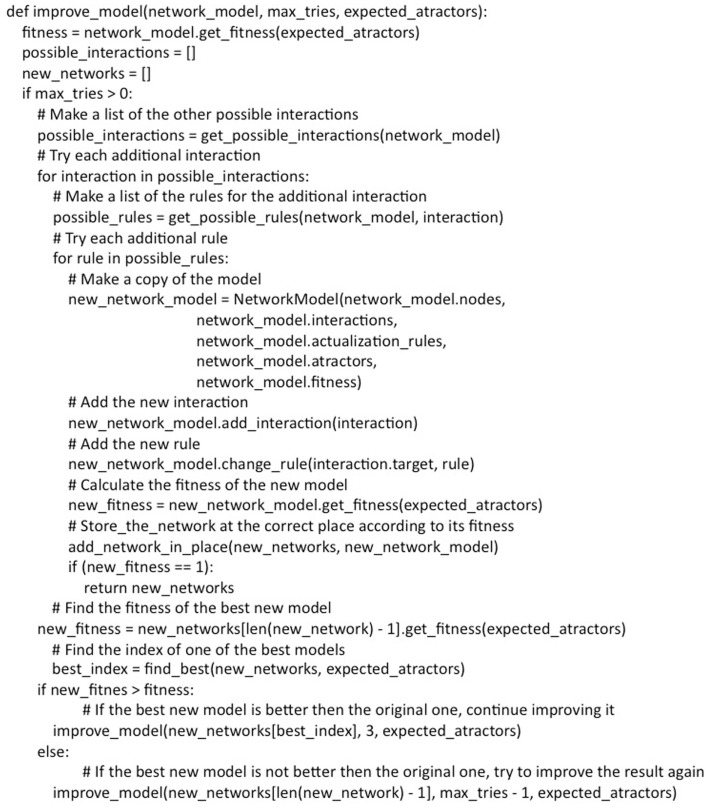
**Pseudocode of the methodology used to incorporate putative missing interactions**.

(1) Generate all putative single missing interactions. The putative missing interactions were those that were not already present in the model and were not contradicted by any available experimental evidence.
(2) Generate all possible BFs of the putative missing interactions maintaining consistency with available biological data using the above procedures.
(3) Test one-by-one all BFs generated and obtain the set of attractors generated with the added interaction.
(4) Select and incorporate into the model the BFs that most improved the model. The criteria to assess if the addition of a regulatory interaction conferred an improvement in the model were, in order of relevance: (a) if the BF increased the number of expected attractors recovered and (b) if the BF reduced the number of non-expected attractors. Here, we defined our expected attractors as the stable gene expression patterns observed experimentally in the RSCN of *A. thaliana* using transcriptional or translational reporter genes. Many genes have oscillatory expression behavior in the root (Moreno-Risueno et al., 2010). However, to our knowledge, none of the genes considered in the updated version of the RSCN GRN have this type of oscillatory expression behavior. Thus, for this particular case, reducing the number of non-expected attractors included eliminating the cyclic attractors.
(5) If more than one BF equally improved the fitness, one BF was randomly selected and added to the model.
(6) After the inclusion of a putative missing interaction, we returned to step 1 unless the model recovered only the expected attractors, or the inclusion of three consecutive BFs did not further improve the model fitness (Figure 4). In Figure 5, we present a pseudocode of the algorithm.

Using the above procedures, which greatly reduced the number of BFs to test (see below), the generation of all the predictions for each model implied testing approximately 100,000 different networks, which is a highly demanding computational process. Thus, we performed the algorithm 10 times, resulting in 10 different models that predicted different putative interactions. We were able to generate 10 different models because more than one BF equally improved the model each time, allowing us to randomly select different BFs. We applied our procedures using the algorithm to the Boolean GRN of the *A. thaliana* RSCN.

### Hypothesis

#### RSCN GRN updated model behavior

Based on available experimental data, we defined nine expected fixed-point attractors (Table 3) for each cell type in the RSCN and some root meristem cell types. Some attractors represented more than one cell type due to lack of experimental data in the model to distinguish among cell types (Figure 1). With the updated RSCN GRN model, we obtained 7 of the 9 expected attractors, 21 attractors without biological meaning in the RSCN context, and 4 cyclic attractors. This result suggests that missing data are yet to be incorporated into the RSCN GRN. Hence, we employed our set of procedures as described above to predict possible missing interactions in the network. The procedures used in each node depended on the type of available data for each gene. In Table 4, we present the procedures used to propose putative missing interactions for each gene, and the experimental information used in each case is provided in Table 1. The self-regulatory loops of nodes with movement must be positive, and hence, we applied procedure 2 in these nodes.

**Table 3:** **Expected attractors**.

CT/G	SHR	miR	JKD	MGP	PHB	SCR	IAA	A/I	WOX	CLE	ACR
CVC	1	0	0	0	1	0	0	1	0	0	0
PVC	1	1	0	0	0	0	0	1	0	0	0
End	1	1	1	1	0	1	0	1	0	0	0
Cor	0	1	1	0	0	0	0	1	0	0	0
LCC	0	0	0	0	0	0	1	0	0	1	1
VI	1	1	0	0	0	0	1	0	0	0	0
CEI	1	1	1	1	0	1	1	0	0	0	0
CLEI	0	1	0	0	0	0	1	0	0	1	1
QC	1	1	1	0	0	1	1	0	1	0	0

*CT, Cell type; G, Gene; CVC, Central Vascular cells; PVC, Periferal vascular cells; End, Endodermis; Cor, Cortex; LCC, Lateral root-cap and columella cells; VI, Vascular initials; CEI, Cortex-endodermis initials; CLEI, Columella and lateral root-cap-epidermis initials; QC, Quiescent center; miR, miRNA165/6; IAA, Auxin; A/I, Aux/IAA; WOX, WOX5; CLE, CLE40; and ACR, ACR4*.

**Table 4:** **Procedures used when adding putative missing interactions in each node**.

	Procedure 1	Procedure 2	Procedure 3	Procedure 4	Procedure 5	Procedure 6
SHR	NO	YES	NO	NO	YES	YES
miR	YES	YES	YES	YES	YES	YES
JKD	YES	YES	YES	YES	YES	YES
MGP	YES	YES	YES	YES	YES	YES
PHB	YES	YES	NO	NO	YES	YES
SCR	YES	YES	YES	YES	YES	YES
Auxin	NO	YES	NO	NO	YES	YES
Aux/IAA	YES	YES	NO	NO	YES	YES
WOX5	YES	YES	YES	NO	YES	YES
CLE40	NO	YES	NO	NO	YES	YES
ACR	YES	YES	NO	NO	YES	YES

#### Predicted putative missing interactions in the RSCN GRN

The combined addition of three new regulatory interactions was sufficient to recover the expected attractors in the different cell types in the RSCN (Figure 3C). Interestingly, these three interactions were also the first ones to appear. No matter which order we included these three interactions, our methodology never proposed any other putative missing interactions until the three were included in the model. This result suggests that these three interactions are fundamental to recover the observed attractors in the RSCN. However, adding these three interactions was not sufficient to eliminate cyclic attractors or several biologically meaningless attractors. In fact, the inclusion of these three interactions always increased the number of cyclic or unexpected attractors. We tried to avoid the increase of cyclic attractors by selecting only networks that simultaneously reduced the number of cyclic attractors and recovered biologically significant attractors. However, this procedure was unsuccessful (data not shown).

Interestingly, the three interactions mentioned above were functionally similar in the 10 replicas of the search process (Figure 3C). The first interaction is a regulation of PHB that restricts its expression domain to the vascular cells. The regulation of PHB was either positive regulation by those nodes with a similar expression domain (e.g., SHR and Aux/IAA) or negative regulation by those genes with a complementary expression pattern (e.g., CLE and ACR4). We postulate that the likely regulator of PHB is a member of the KANADI (KAN) gene family. *KAN* genes were not included in this GRN model because no connections with any node of the RSCN GRN in the root have been documented yet; however, KAN genes have antagonistic roles with PHB in the shoot and have a complementary expression pattern to *PHB* in the root (Figure 3C; Hawker and Bowman, 2004; Izhaki and Bowman, 2007).

The second interaction is a regulation over WOX5. Almost all the networks predicted that this regulation should be a feedback loop. The WOX5 loop could be direct or indirect, as well as positive or negative (Figure 3C). Interestingly, some experimental and theoretical evidence supports the existence of such a loop through the auxin signaling pathway (Gonzali et al., 2005; Azpeitia et al., 2010), and our results suggests that this feedback look could exist. However, contradictory experimental evidence has been reported on this issue. Positive regulation of *WOX5* by auxin has been reported (Gonzali et al., 2005), while other data suggest that auxin negatively regulates *WOX5* (Ding and Friml, 2010). Based on the interactome analysis, our model proposes that ARF activators are positive regulators, while ARF inhibitors are negative regulators of WOX5; therefore, this model includes both possibilities. With this model, we predict that *WOX5* should negatively regulate the auxin signaling pathway. Our model assumed that ARFa was always capable of promoting *WOX5* expression, as proposed by Vernoux et al. (2011). However, if the results that the negative regulation of *WOX5* by the auxin signaling pathway is stronger than the positive regulation, as Ding and Friml (2010) proposed, then the regulation of the auxin signaling pathway by WOX5 should be positive. The third interaction is a positive regulation of *JKD* by MGP (Figure 3C). The interplay between JKD, MGP, SCR, and SHR is complex (Welch et al., 2007; Ogasawara et al., 2011), and our simulations suggest that additional possible regulatory mechanisms should be considered, highlighting the ability of our procedures to detect probable missing data.

After the inclusion of 11–15 interactions, the performance of the resulting GRN models no longer improved. After this point, almost all models reduced both the number of cyclic and biologically meaningless attractors to three. Interestingly, some interactions were present in several of the 10 final models. Specifically, the most common interactions were: (1) inhibition of *SHR*, (2) activation of *SHR* by PHB, (3) negative regulation of auxin by PHB, and (4) negative regulation of *CLE40* by Aux/IAA or SHR. The BFs in the original model and the 10 models with putative missing interactions are available in Supplementary Material, and all putative missing interactions predicted by the whole set of simulations are available in Supplementary Material. Importantly, all putative missing interactions that were proposed using our procedures were biological meaningful, did not contradict previous experimental data, and are experimentally testable. Our results indicate key gaps in the data concerning the regulation of nodes in the RSCN GRN. Unraveling how these genes are regulated will be fundamental to our understanding of how the RSCN is maintained. However, our work already suggest possible nodes and missing interactions needed to obtain a sufficient model of RSCN patterning.

#### Efficiency of the procedures

The reduction of possible BFs obtained with our procedures is astonishing. For example, using procedures 4, 5, and 6 together on all regulatory genes, no matter the number of regulators, always resulted in 4 possible BFs. Using these procedures on all nodes is equivalent to reducing all nodes to 1, which represents a dimer or protein complex. This result was important for the RSCN GRN because SHR and SCR form a dimer that is only functional if both proteins are present (Cui et al., 2007). Thus, the TGENs of the dimer used procedure 4. Using this procedure, we only needed to test tens of BFs from the over four billion possible BFs of JKD and MGP. Because of this reduction, we only tested ≈3,000 of ≈8 × 10^9^ BFs to generate the first set of possible BFs in the model.

The efficiency of the use of procedures 1, 2, and 3 and combinations of the procedures needs to be formally analyzed in future studies. However, previous work demonstrated that using procedures 5 and 6 reduces the number of BFs from 16 to 8, 256 to 72, and 65,536 to 1882 for a node with 2, 3, and 4 RGENs (Raeymaekers, 2002), respectively. This previous study suggests that using combinations of our procedures should be able to reduce the number of BFs further, making the combinations useful in the prediction of putative missing interactions. The total reduction is completely dependent on the quantity and quality of the available experimental data, which will determine the procedures to use.

#### Usefulness of the procedures

In addition to the utility of the procedures for predicting putative missing interactions, we detected other important uses of the procedures. The first important use is evident when the experimental data are only sufficient to use procedure 1, or procedure 1 combined with procedures 5 or 6. In this case, positive regulators can be negative regulators, and *vice versa*. Thus, when we apply procedure 1 to predict a putative missing interaction, regulatory genes can change their sign of regulation. This result is important because it demonstrates that some experiments commonly used to infer gene regulatory interactions are not sufficient to assure the sign of regulation (see Appendix 2 in the Appendix). We can use procedure 1 to detect, and later test experimentally, if a positive regulator was identified as a negative regulator, and *vice versa*. We detected a second use when applying any single procedure or combination of procedures, except procedure 4. In this case, single proteins within protein complexes can act as independent units. The proteins are not necessarily required to act as independent units; however, this result helps us detect cases where proteins can, or need to, be substituted in a protein complex or when the proteins can regulate the activity of a TGEN as independent units or as units of different protein complexes. These predictions can be experimentally validated (see Appendix 2 in the Appendix).

## Discussion and Conclusion

All, or most, GRN models are incomplete because they likely lack components or interactions due to incomplete experimental data and computational limitations. However, even for small BNs, the detection of such missing data is difficult because the number of possible BFs and topologies describing the interactions rapidly becomes overwhelming as the number of nodes and interactions being considered increases. We have proposed here a set of procedures that greatly reduce the number of possible interactions and enable the detection and prediction of biologically meaningful, putative missing interactions, while maintaining congruence with available and already incorporated experimental data. Our procedures were designed to maintain congruence with different types of experimental data and greatly reduce the number of possible BFs to be tested (≈3,000 out of over ≈8 × 10^9^ in the example of the RSCN GRN). Importantly, we tested our procedures with smaller network motifs to assure that our procedures worked as expected before testing the procedures on the RSCN GRN.

The magnitude of the reduction in the putative BFs greatly depends on the quality of the available data and the nature of the interactions. Depending on the quality of the data, different BFs are generated. Importantly, our procedures demonstrate that some experiments that are usually used to determine the sign of a regulatory interaction are not reliable or are not adequate to uncover the actual interaction in diverse contexts (Lewontin, 2000). Similarly, some experiments that indicate the necessity of a protein complex for the expression of a TGEN are also not reliable. Furthermore, these situations are frequently not intuitive, and the procedures put forward here enable the detection of the circumstances under which such mistaken inferences can occur. Once the circumstance involved is known, we can easily design experiments to dismiss such situations. However, if we have enough experimental data to confirm the sign of the regulation or the presence of a complex, then we can use the proposed procedures to maintain these experimental data contained in the BF without change.

Using these procedures, we have designed an evolutionary algorithm to systematically predict possible missing interactions, and we have applied this approach to the *A. thaliana* RSCN GRN. Our work provides concise predictions concerning additional interactions and a novel RSCN GRN architecture that could be tested experimentally. Importantly, our work has identified three additional key interactions that could be studied: (i) regulation of *PHB* to maintain its expression pattern in the vascular cylinder, (ii) a feedback loop regulating *WOX5*, and (iii) positive regulation of *JKD* by MGP. However, we were not able to recover a network that attained only the experimentally observed gene configurations without the presence of unobserved attractors. Additional missing nodes, such as SCZ (ten Hove et al., 2010) or *root growth factors* (Matsuzaki et al., 2010), may be required to recover only the observed set of configurations. Because we were interested in finding missing interactions within already connected RSCN genes, we decided to dismiss genes that were unconnected from those included in this work, such as SCZ. Another possible explanation for why we never obtained only the expected attractors is that we only included putative missing interactions one-by-one. Including two or more putative missing interactions each time could change the results due to combinatorial effects. As explained previously, the computational demand for including one interaction can be very large. Hence, the computational demand of adding interactions simultaneously rapidly explodes. However, we believe that our approach provides a formal, systematic framework to postulate novel hypotheses concerning the way genes interact. For small networks, testing the effect of adding multiple interactions is possible.

There still are several improvements that could be done to the procedures. The inclusion of a genetic algorithm would allow a search for missing interactions not only one-by-one but also by sets of putative missing interactions at one time. Optimizing with Binary Decision Diagrams (BDDs) or more efficient algorithms could also allow for testing of more than one interaction. A way to simplify the use of our procedures is to incorporate them into an existing dynamic network analyzer (e.g., Arellano et al., 2011). Procedures that use information of the GRN topology or about the effect of how genes in the networks indirectly affect other genes should further reduce the number of BFs generated when we add putative missing interactions. For example, we could already know that in the RGEN1 loss-of-function mutant, TGEN = 0, while RGEN3 = 1, but that RGEN3 is not a TGEN of RGEN1. In this case, if we add RGEN3 as a putative missing regulator of TGEN, we will know that in the new rows of the TGEN’s truth table where RGEN1 = 0, TGEN expression value will be 0 when RGEN3 = 1 and TGEN’s expression value will be unknown when RGEN3 = 0. The use of this type of data for the generation of more procedures was not explored in this work, but should be addressed in future research.

The fact that we used BNs in this work implies both strength and weakness. BNs allowed us to exhaustively test all the possible GRNs generated by adding putative missing interactions; however, BFs are unable to represent certain types of regulatory interactions, such as those implying fine-tuning modulations of regulatory activity. An improvement to our procedure would involve extending the procedures to consider multivalued discrete networks that can better evaluate more circumstances, although this method would also increase the computational demand.

Finally, given that the methodology used in this work can be applied to any BN, we believe that this type of exploration could help guide experimental research not only of biomolecular GRNs but also of any biological, physical, or theoretical system that can be formalized as a Boolean interaction network. For example, this methodology can be used to study the constraints that a given network topology imposes on attractor evolvability. However, formal mathematical demonstrations should be performed first.

## Conflict of Interest Statement

The authors declare that the research was conducted in the absence of any commercial or financial relationships that could be construed as a potential conflict of interest.

## Supplementary Material

The Supplementary Material for this article can be found online at http://www.frontiersin.org/Plant_Systems_Biology/10.3389/fpls.2013.00110/abstract

## Figures and Tables

**Figure A1:**
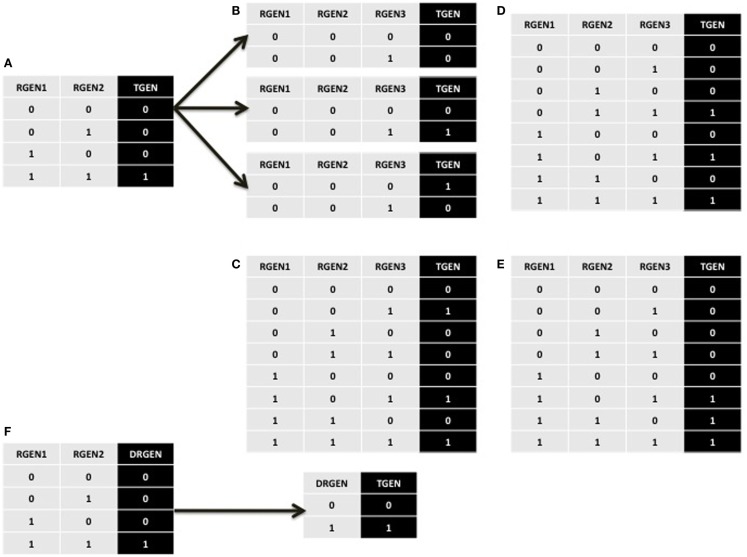
**Partial and complete truth tables describing the procedures explained in the main text**. **(A)** Equivalent truth table for the logical statement “TGEN, RGEN1, AND RGEN2.” **(B)** The three possibilities for the first line of the truth table if a putative missing interaction is added. Each line describes one experiment. **(C)** A truth table where the original positive regulation of TGEN by RGEN2 observed in **(A)** changes to a negative regulation. **(D)** A truth table where the original AND operator between RGEN1 and RGEN2 observed in **(A)** changes to an OR operator indicating that they do not need to form a dimmer. **(E)** A truth table where RGEN2 can be substituted by RGEN3 from the RGEN1-RGEN2 dimer. **(F)** When two RGENs can only act as a dimer, and none of them can be substituted in the dimer, we can create a node that represents the dimer. The truth table observed in **(A)** where RGEN1 and RGEN2 regulate TGEN activity is substituted by a new truth table where the activity of the RGEN1-RGEN2 dimer is incorporated into a single node.

**Figure A2:**
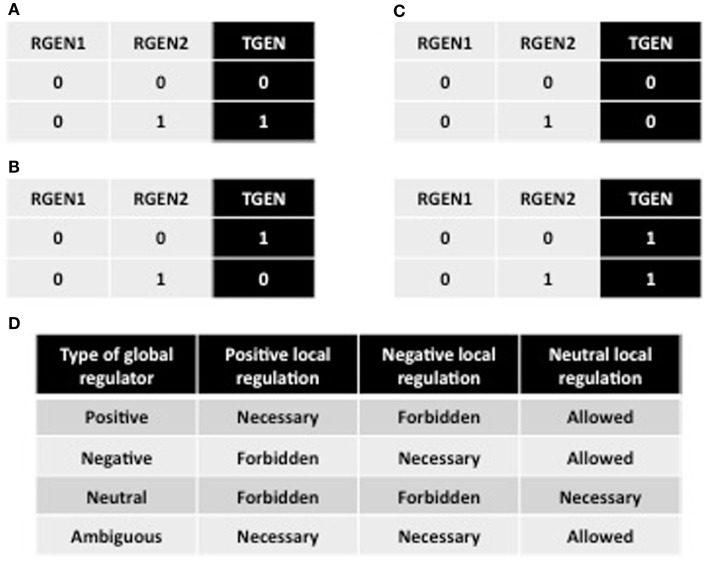
**Partial truth tables representing local regulations and definitions of global regulators**. A portion of the truth tables where RGEN2 acts as a local positive **(A)**, negative **(B)**, and neutral regulator **(C)**. **(D)** The definitions of global regulators based on local regulator definitions. The table shows which types of local regulations are necessary, allowed, or forbidden for each type of global regulation.

**Figure A3:**
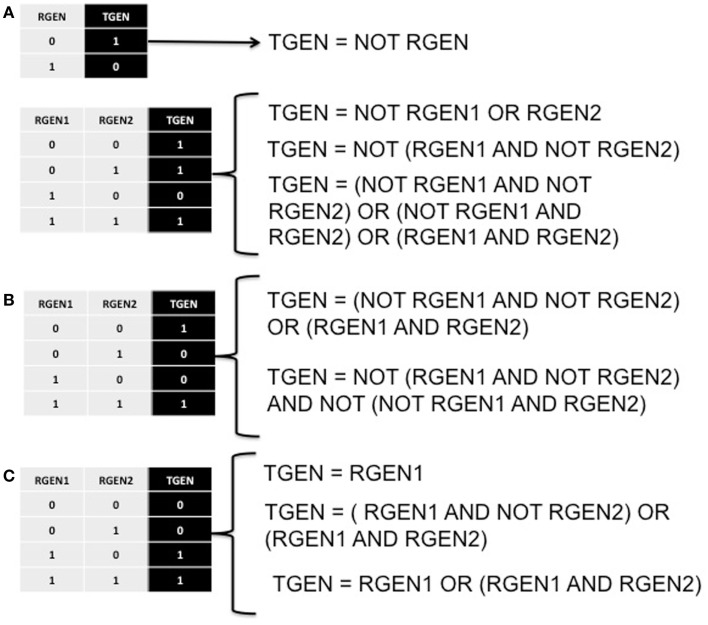
**Truth tables and equivalent logical statements for global negative, ambiguous, and neutral regulator definitions**. **(A)** Two examples of a negative global regulator. As observed in the first example, the regulator can only be expressed with a NOT logical operator in the logical statement. In the example below, equivalent logical statements where RGEN1 acts as a negative and as an ambiguous regulator can be used. However, we can observe that if RGEN1 is labeled as a negative global regulator, we can always express RGEN1 in a logical statement with a NOT logical operator. **(B)** An example of ambiguous global regulators. In this example, the regulators can only be expressed as ambiguous regulators using a NOT logical operator in one part of the sentence and omitting the NOT operator in the other part of the sentence. **(C)** A neutral global regulator. The logical statement can be written without including the neutral global regulator (RGEN2). However, we can use an equivalent logical statement to include RGEN2 as a positive, negative, or neutral regulator. Neutral global regulators can always be removed from the logical statement.

**Figure A4:**
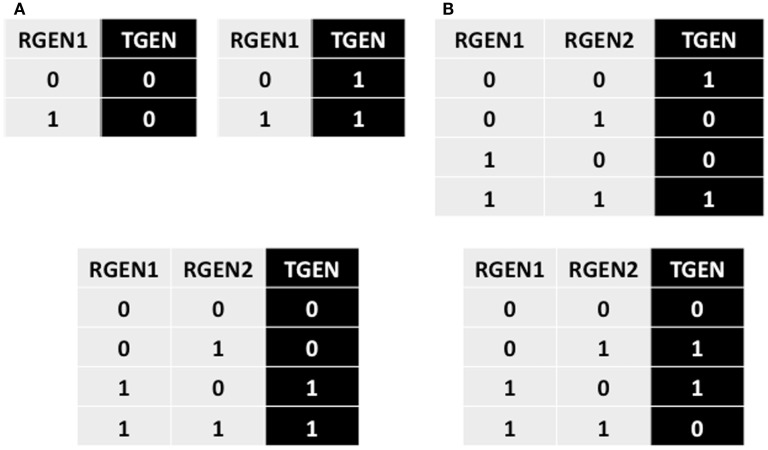
**Truth tables with global neutral and ambiguous regulations of TGEN**. **(A)** Truth tables with global neutral regulations of TGEN by RGEN1 or RGEN2. In the top two truth tables, we observe that the expression of TGEN does not change independently of the expression value of RGEN1. In the truth table below, we observe that the change in TGEN’s expression value depends only on the value of RGEN1 but not on the expression value of RGEN2. **(B)** Truth tables with global ambiguous regulation of TGEN by RGEN1 and RGEN2. In both truth tables, TGEN’s expression value is positively and negatively regulated by RGEN1 and RGEN2 indicating that both factors are ambiguous regulators of TGEN.

## Appendix 1

### Auxin signaling pathway reduction

In this section, we first briefly describe the auxin signaling pathway and then explain the analysis of the ARF-Aux/IAA interactome using MixNet, a publicly available software program designed for structural network analysis. For the interested reader a more detailed explanation of MixNet can be found in this reference (Picard et al., 2009).

In the auxin signaling pathway, the Aux/IAA genes repress the transcriptional activity of the ARFs by forming heterodimers. ARFs can be classified based on their transcriptional activity; ARFs 5, 6, 7, 8, and 19 are transcriptional activators (ARFa), while all other ARFs are putative transcriptional inhibitors (ARFi; Guilfoyle and Hagen, 2007). ARFa and ARFi compete for the same TGENs (Ulmasov et al., 1999). The SCF^TIR1^ ubiquitin ligase complex promotes Aux/IAA degradation in the presence of auxin, releasing ARFs from Aux/IAA inhibition. Once ARFs are released from Aux/IAA inhibition, ARFs are able to perform their transcriptional activity.

Recently, Vernoux et al. (2011) published an ARF and Aux/IAA interactome and analyzed how these proteins interact in the shoot and whole seedling using MixNet (Picard et al., 2009). MixNet uses a probabilistic clustering method that allows for the identification of structural connectivity patterns. Because MixNet relies on an algorithm that does not make any *a priori* assumptions about network structural properties, MixNet allows a blind search of highly or poorly interconnected groups of nodes. MixNet considers that nodes can be divided into *Q* connectivity classes, with *Q* being unknown. As a result, MixNet returns to the user a value α, which is the proportion of each group, and π, the connectivity of the groups. Finally, if *Z*_iq_ = 1, then node *i* belongs to class *q*. Hence, MixNet describes the network topology using connectivity probabilities among nodes, such that π_q_p represents the probability for a node from group *q* to be connected to a node from group *p* (Picard et al., 2009). Model selection in MixNet can be performed based on the ICL and incomplete data likelihood criteria.

Rademacher et al. (2011) reported the expression patterns of ARFs in the root, while Brady et al. (2007) created a high-resolution expression map of the root that included the Aux/IAA gene family. We defined the Aux/IAA and ARF genes that are expressed in the root based on these previous studies and analyzed their interactome reported previously (Vernoux et al., 2011). Based on these considerations, we considered the following ARF and Aux/IAA genes:

ARFs:
ARF1, ARF2, ARF5, ARF6, ARF7, ARF8, ARF9, ARF10, ARF16, and ARF19.

Aux/IAAs:
IAA1, IAA2, IAA3, IAA5, IAA6, IAA7, IAA8, IAA9, IAA10, IAA11, IAA12, IAA13, IAA14, IAA16, IAA17, IAA19, IAA20, IAA27, IAA28, IAA29, IAA30, IAA32, IAA21, and IAA33.

We applied the MixNet algorithm for *Q* = 1–15 clusters and used the ICL criterion for model selection. As Vernoux et al. (2011), reported, based on the ICL criterion, the MixNet analysis favors four clusters. However, this solution is only valid for a large N; therefore, we used the three cluster (*Q* = 3) solution as reported in Vernoux et al., 2011. This solution implies that the Aux/IAA and ARF proteins are divided into three different groups. The first group was comprised mostly of Aux/IAA proteins, which interact among themselves, and ARFa. The second group was mostly comprised of ARFa, which interacts only with Aux/IAA. The third group was mostly comprised of ARFi, which does not interact with any other group. This model of the auxin signaling pathway is very general; however, as more information becomes available, a more detailed auxin signaling pathway will be possible.

The probability matrix π, the nodes comprising each cluster and the interactions among the Aux/IAA and ARF proteins extracted from the work of Vernoux et al. (2011) are given below.

π Matrix:
0.110916, 0.0848193, 0.275044.
0.0848193, 0.745456, 0.856257.
0.275044, 0.856257, 0.240615.
Cluster 1:
ARF1, ARF2, ARF10, ARF16, ARF18, IAA6, IAA11, IAA29, IAA31, IAA32.
Cluster 2:
IAA1, IAA2, IAA3, IAA7, IAA8, IAA10, IAA12, IAA13, IAA14, IAA16, IAA17, IAA19, IAA20, IAA27, IAA28, IAA30, IAA33.
Cluster 3:
ARF5, ARF6, ARF7, ARF8, ARF9, ARF19, IAA5, IAA9.

The only nodes that do not behave as expected were IAA5, IAA11, IAA29, IAA31, and IAA32, which belong to cluster 1, and IAA5 and IAA9, which belong to cluster 3. We expected these nodes belonged to cluster 2.

The topology of the auxin signaling pathway according to this result eliminated the Aux/IAA-ARFi interaction. In this model, ARFi modulates the ARFa response once ARFa proteins are released from Aux/IAA inhibition. However, in the presence of high auxin concentration, ARFa always activates its TGENs (Vernoux et al., 2011). Boolean models cannot represent the degree of the response due to the ARFa/ARFi ratio. Consequently, we eliminated ARFi from the GRN, resulting in a linear pathway where ARFa activity is only regulated by Aux/IAA in the GRN. Moreover, ARFa proteins are constitutively expressed in all cells of the root meristem, including the RSCN. Hence, ARFa does not need to be included in the GRN because its activity is equally represented by the auxin response that is triggered when the auxin concentration promotes Aux/IAA degradation. Consequently, we reduced the auxin signaling pathway to the auxin and Aux/IAA nodes.

## Appendix 2

### Detailed description of the procedures

In this section, we describe how we designed the 6 procedures used to infer putative missing interactions in the RSCN GRN. As explained in the main text, we used truth tables to analyze how the experimental data are contained in the BFs. However, we should be able to write a negative regulator with a NOT operator and a protein–protein interaction with an AND operator. For this reason, we will continue to use logical statements whenever useful in this section.

#### Procedure 1: adding missing links while maintaining congruence with available experimental data that can be represented by a single row in the truth table

Let us assume a TGEN is expressed when RGEN1 and RGEN2 are both expressed, which is represented by the last row of the truth table in Figure A1A. In addition, we will assume that TGEN is not expressed in the single loss-of-function RGEN1 and RGEN2 mutants, represented by the second and third rows of the truth table, respectively. Finally, we will suppose that TGEN is not expressed when both RGEN1 and RGEN2 are not present, and this scenario is represented in the first row of the truth table. In this example, each experiment is formalized as a single row of the truth table. Now, how can a third RGEN3 be added to the TGEN’s BF without contradicting the previously incorporated experimental data? Knowing that each row of the truth table represents one experimental result can help us. As observed, in the first row of the truth table without the addition of RGEN3, the TGEN expression state is *0*. To maintain consistency with these data in the truth table once RGEN3 is added, the TGEN expression state must remain at *0* in at least one of the truth table’s rows where RGEN1 and RGEN2 are not expressed. The possible rows that fit these criteria are shown in Figure A1B. To maintain consistency with the experimental data contained in the other rows, we perform the same analysis of all other rows in the truth table. Thus, in this example, we must maintain at least one *0* for the expression value of TGEN whenever RGEN1 or RGEN2 are not expressed and a value of *1* whenever RGEN1 and RGEN2 are both expressed when RGEN3 is added. Hence, when each experiment is represented by a single row in a truth table, we need to maintain at least one *0* as the TGEN expression value under the conditions where no expression of TGEN was experimentally observed, and at least one *1* under the conditions where TGEN expression was experimentally observed; this process maintains consistency with the previously incorporated experimental data when putative missing interactions are added to the truth table. This procedure generates many possible BFs once we add a putative missing interaction. Nevertheless, the procedures is useful when an experiment is contained in a single row of a truth table. A common use of this procedure occurs when the only available experimental data are single and multiple gain- and loss-of-function mutants.

#### Procedure 2: adding missing links while maintaining the sign of the regulation

In Figure A1C, we present a truth table that we can be generated using procedure 1. One logical statement that can represent this function is “TGEN = RGEN3 AND (RGEN1 OR NOT RGEN2).” In this logical statement, RGEN2 changed from being a positive to a negative regulator of TGEN. However, we need to define positive and negative regulation in the BF context to assure that this change occurred.

If a RGEN positively regulates the TGEN, then we should observe in the truth table that when RGEN is ON, TGEN should also be ON, at least under one condition. Here, we defined a *condition* as the set of states where all RGENs of a TGEN have a fixed expression value, except the RGEN for which we are analyzing the sign of its regulation. Hence, we defined RGEN as a *local positive regulator* of TGEN, when TGEN and RGEN expression states are the same under identical conditions (Figure A2A). Conversely, we defined RGEN as a *local negative regulator* when TGEN and RGEN expression values are different under the same conditions (Figure A2B). Finally, we defined RGEN as a *local neutral regulator* of TGEN if the latter does not change its expression value irrespective of its regulator state (Figure A2C).

An absolute positive regulator of a TGEN should never be able to act as a local negative regulator of the target. However, this rule does not mean that a positive RGEN must activates the TGEN under all conditions. Thus, we defined a global positive regulator as a RGEN that acts as a local positive regulator or as a local positive and local neutral regulator. A global negative regulator acts as a local negative regulator or as a local negative and local neutral regulator. However, a global neutral regulator only acts as a local neutral regulator. Finally, we defined ambiguous global regulators as those RGENs that act as local positive and local negative regulators or as local positive, local negative, and local neutral regulators (Figure A2D).

A node labeled as a negative regulator according to our global regulator definitions can always be expressed with a NOT logical operator in the logical statement; however a negative regulator cannot be represented as a neutral regulator and may not be represented as a positive or ambiguous regulator as observed in Figure A3A. The same definitions apply to positive nodes. An ambiguous node according to our global regulator definitions can always be expressed as an ambiguous regulator, but not as a positive, negative, or neutral regulator (Figure A3B). Finally, a truth table with a neutral global regulator can always be written as a logical statement without the inclusion of the regulator, even when the regulator can be included in another equivalent logical statement (Figure A3C). Thus, our global regulator definition assures that a regulator labeled as negative, positive, neutral, or ambiguous can be expressed in a logical statement with the correct logical operator.

Consequently, to verify the sign of the regulation, we use our global regulator definitions. Applying the global definitions, we can analyze the truth table in Figure A1C and observe that RGEN2 is a local negative and local neutral regulator of TGEN. Therefore, RGEN2 is a global negative regulator. In contrast, in the original truth table without RGEN3 added (Figure A1A), RGEN2 acted only as a local positive regulator; hence, RGEN2 was a global positive regulator. Thus, when we use procedure 1 to generate BFs, RGENs can change their sign of regulation. This result indicates that some experiments, such as the ones used in our example and which are commonly used to infer gene regulatory interactions, are not sufficient to assure that a RGEN is a positive or negative regulator. Importantly, the use of procedure 1 can identify the circumstances under which a positive regulator can be falsely identified as a negative regulator, and *vice versa*.

In some occasions, a RGEN is known to be either a positive or a negative regulator of its TGEN. For example, high quality experimental data, such as chromatin immunoprecipitation, might indicate that RGEN1 and RGEN2 are direct positive regulators of TGEN. These data will not change the truth table in Figure A1A. We can use our definitions to include putative missing interactions without changing the experimentally observed sign of the regulatory interaction. If RGEN is a known negative regulator, we use procedure 2 to exclusively generate all the BFs where RGEN acts as a negative local regulator or as a negative and neutral local regulator.

Using our global regulator definitions in procedure 2, the RGEN regulation sign can be expressed in the desired manner in a logical statement (e.g., with a NOT if we want a negative regulator). Thus, using our procedure, the RGEN regulatory sign can be expressed in a way that maintains consistency with the sign of regulation reported experimentally.

#### Procedures 3 and 4: adding missing links while maintaining documented protein–protein interactions

In another scenario, a yeast two hybrid assay, or other method, confirmed that RGEN1 and RGEN 2 are not only positive regulators of TGEN, but RGEN1 and RGEN2 also interact at the protein level and form a dimer. In our example, when we add RGEN3, procedures 1 and 2 can generate the truth table observed in Figure A1D. One logical statement that can represent this function is “TGEN = RGEN3 AND (RGEN1 OR RGEN2).” Using this logical statement, RGEN1 and RGEN2 do not need to act as a dimer. However, we need to define the expression of a dimer in a BF before assuring the last statement.

Some transcriptional regulators act as dimers or more complex multimers. A TGEN activity is independent, locally, and globally, of a global neutral RGEN. However, if two RGENs function as a dimer, neither RGEN1 nor RGEN2 can act as local neutral regulators in the dimer. Anyhow, in the truth table in Figure A1D, RGEN1 and RGEN2 are local neutral regulators in both conditions where RGEN1 and RGEN2 could form a dimer, which is what we do not want that happens if we want to maintain the dimer functionality. Using the sign definitions defined previously, we can generate a procedure to generate BFs that maintain the dimer functionality, namely procedure 3. In this procedure, to maintain the dimer functionality, we need to verify that at least one local non-neutral regulation is specified for each RGEN in the same row, and in this row they must be capable to act as a dimmer (i.e., have a *1* expression value). Variations to this procedure can be used to maintain different types of interactions among regulators.

Finally, using procedure 3, we can generate the truth table observed in Figure A1E. One logical statement that can represent this function is “TGEN = RGEN1 AND (RGEN2 OR RGEN3).” This statement indicates that RGEN3 can substitute for RGEN2 in the dimer. However, the presence of a RGEN in a dimer can sometimes be necessary to regulate the expression of a TGEN. In this situation, the dimer RGEN1-RGEN2 is only functional when both proteins are together, and none of them can be substituted. The simplest way to maintain these data is by creating a new node, namely DRGEN (to indicate a dimer of RGENs) that represents the complex formed by RGEN1 and RGEN2. Subsequently, the TGEN truth table can be redefined in terms of DRGEN (Figure A1F). Using this method, none of the RGENs that form a complex can be substituted.

Is important to note that these first four procedures only use the available information about how RGENs regulate their TGENs. This implies that we do not include information about how RGENs affect each other or about the network topology nor any kind of partial information regarding possible indirect regulation of TGEN by RGENs. Thus, the procedures could be improved if we include data about the effect of RGENs on another RGENs of the GRN that are not their TGENs or if we use information about the network topology. The use of this data could reduce even more the number of BFs generated when we add a putative missing interaction, but will complicate the algorithm design and greatly increase the number of procedures. Because we did not include this kind of data to design the procedures, the number of possible BFs could be overestimated. However, the algorithms and procedures design is simpler.

#### Procedures 5 and 6: adding missing links without ambiguous regulators and incorporating independent TGEN activity

While the procedures described above were dependent on a set of experimental data that are available when reconstructing a truth table with added interactions and/or nodes, these two additional procedures stem from the limits of the Boolean formalism, and we propose these procedures to simplify the interpretation of the predicted missing interactions and reduce the number of BFs generated when we add a putative missing interaction.

The activity of a TGEN may be independent of the activity of one or more of its RGENs. For example, in the truth tables in Figure A4A, which are represented with the logical statements “TGEN = 0,” “TGEN = 1” and “TGEN = RGEN1,” TGEN activity is not affected by RGEN1 in the first two and is independent of RGEN2 in the third one. Some of these cases appear because certain gene regulations cannot be represented as BFs due to missing data or the nature of the interactions. For example, the role of some proteins whose function is to modulate the activity of other proteins cannot be represented as a BF. Consequently, BFs where one or more of the RGENs were global neutral regulators were not considered because these BFs indicate that the TGEN activation state is independent of one or more RGEN or the RGENs regulatory effect cannot be represented with a Boolean formalism. We refer to this procedure as procedure 5.

Finally, we decided not to consider BFs where one or more RGENs were ambiguous global regulators (Figure A2D; see example of an ambiguous regulator in Figure A4B). This is a simplifying assumption, because some genes are indeed ambiguous regulators. However, some authors propose that a dual regulatory role is not common (Davidson, 2001), and a biological interpretation is difficult to provide in cases where the number of ambiguous RGENs increases in the BF. Constraining the BF to only those with unambiguous global regulators greatly reduces the number of BFs (e.g., only 1882 of 65,536 for four regulators; La Rota et al., 2011). We refer to this procedure as procedure 6. Importantly, the use of these last two procedures has been discussed and analyzed previously, demonstrating its utility and biological importance (Raeymaekers, 2002).

Using our set of procedures, we can incorporate putative missing interactions that are congruent with the available experimental data and imply novel predictions without contradicting previously available experimental data. The methodology is explained above.
