# Supplementary material for: Finding Missing Interactions of the Arabidopsis thaliana Root Stem Cell Niche Gene Regulatory Network
Source: Front Plant Sci. 2013 Apr 30;4:110. doi: 10.3389/fpls.2013.00110 (PMC3639504; doi:10.3389/fpls.2013.00110)
Supplement: Supplementary file 1 [file Data_Sheet_1.DOC]

**Intermediary nodes.**

Intermediary nodes that integrated the activity of two RGENs over its TGEN could appear as a consequence of procedure 4 or as a way to continue adding putative missing interactions over a TGEN with already 4 RGENs.

In our initial model we started with three intermediary nodes. The first one was conformed by SHR and SCR. These proteins form a dimer that promotes the expression of hundreds of genes (Sozzani et al., 2011). The absence of one of them in the dimer hinders the activity of the other (Cui et al., 2007). Hence, we use *procedure 4* and use an intermediary node, namely SYS (for SHR & SCR), to represent the SHR-SCR dimer (Supp Fig 1 and Supp Mat 4).

In our model, *WOX5* expression was initially regulated by 4 RGENs, namely, CLE40, ACR4, Aux/IAA and PHB. In order to be able to add putative missing interactions over WOX5, we integrated CLE40-ACR4 in a node (CYA), and Aux/IAA-PHB in other (PYI5; Supp Fig 1 and Supp Mat 4).

As the number of putative missing interactions added increased, we continue generating intermediary nodes in genes that reached 4 RGENs. These new intermediary nodes were dependent on the BFs selected after the addition. The BFs (including the BFs for the intermediary nodes) of the initial GRN and the GRNs with added interactions are available in Supp Mat 4.

**6. Supplementary References**

Cui,H. *et al*. (2007). An evolutionarily conserved mechanism delimiting SHR movement defines a single layer of endodermis in plants. Science. 316:421-5.

Sozzani,R. *et al*. (2010). Spatiotemporal regulation of cell-cycle genes by SHORTROOT links patterning and growth. Nature. 466:128-32.
