# Supplementary material for: Finding Missing Interactions of the Arabidopsis thaliana Root Stem Cell Niche Gene Regulatory Network
Source: Front Plant Sci. 2013 Apr 30;4:110. doi: 10.3389/fpls.2013.00110 (PMC3639504; doi:10.3389/fpls.2013.00110)
Supplement: Supplementary file 2 [file Data_Sheet_2.DOC]

BFs of the initial model, and BFs of models after the new interactions were added.

Logical rules of the initial RSCN GRN:

SHR = SHR

SCR = SYS | JKD

JKD = (JKD | SYS) & ! PHB

MGP = JKD & ! (SYS & MGP) | SYS & ! JKD & ! MGP

miRNA165 = SYS | miRNA165

PHB = ! miRNA165

Auxin = Auxin

IAA5 = ! Auxin

WOX5 = PYI5 & ! CYA

CLE = CLE

ACR = CLE

*SYS = SHR & SCR

*CYA = CLE & ACR

*PYI5 = ! PHB & ! IAA5

* indicate intermediary nodes

Logical rules of the RSCN GRN after adding putative missing interactions:

**Model1**

SHR = ( SHR | ! IAA5 ) & ! CLE

SCR = SYS | JKD

JKD = (SYS & JKD | & MGP) & ! PHB

MGP = (JKD | SYS) & ! MGP | JKD & CYA

miRNA165 = SYS | miRNA165

PHB = ! miRNA165 & ! CYA

Auxin = Auxin & ! PHB

IAA5 = ! Auxin

WOX5 = PYI5 & MGP & ( ! CYA | WOX5 )

CLE = CLE & ! JKD & ! IAA5

ACR = ! ( ! CLE & ! CYA )

*SYS = SHR & SCR

*CYA = CLE & ACR

*PYI5 = ! PHB & ! IAA5

**Model 2**

SHR = ( SHR | ! IAA5 ) & ! CYA

SCR = ( SYS | JKD ) & ! CYA

JKD = MGP & ( SYS | ! PHB ) | SYS & JKD & ! PHB

MGP = SYM | JYA

miRNA165 = SYS | miRNA165

PHB, ! miRNA165 & ! CYA

Auxin = ! SHR | Auxin & SCR

IAA5 = ! ( Auxin & ! WOX5 )

WOX5 = PYI5 & ! CYA & JKD

CLE = CLE & ! IAA5

ACR = CLE & Auxin

*SYS = SHR & SCR

*CYA = CLE & ACR

*PYI5 = ! PHB & ! IAA5

*JYA = JKD & ! Auxin

*SYM = SYS & ! MGP

**Model 3**

SHR = ( SYI & ! CYA ) | PHB

SCR = SYS | JKD

JKD = SYS & JKD & (! PHB | MGP) | ! PHB & MGP

MGP = JKD & ! (SYS & MGP) | SYS & ! JKD & ! MGP

miRNA165 = (SYS | miRNA165) & ! JKD | SYS & miRNA165

PHB = ! miRNA165 & (! CYA | IAA5)

Auxin = Auxin & ! PHB & ! IAA5

IAA5 = ! Auxin

WOX5 = MGP & (CYA | ! WOX5 & PYI5)

CLE = CLE & ! WOX5 & ! IAA5

ACR = CLE

*SYS = SHR & SCR

*CYA = CLE & ACR

*PYI5 = ! PHB & ! IAA5

*SYI = SHR | ! IAA5

**Model 4**

SHR = JKD | IAA5 & ( ! CYA | ! SHR)

SCR = JKD & (MGP | WOX5) | SYS & (MGP & WOX5)

JKD = SYS & JKD & (! PHB | MGP) | (SYS & MGP) | (! PHB & MGP)

MGP = JYI | SYM

miRNA165 = SYS | miRNA165

PHB = ! miRNA165 & ( ! CYA | PYI52 )

Auxin = Auxin

IAA5 = ! Auxin & ! CYA

WOX5 = PYI5 & ! CYA & WOX5

CLE = (CLE | ! IAA5) & ! SHR

ACR = CLE & Auxin

*SYS = SHR & SCR

*CYA = CLE & ACR

*PYI5 = ! PHB & ! IAA5

*JYI = JKD & IAA5

*SYM = SYS & MGP

*PYI52 = PHB & IAA5

**Model 5**

SHR = ! CYA & (SHR | ! IAA5) | PHB

SCR = (SYS | JKD) & ! CYA

JKD = SYS & (! PHB | MGP) | ! PHB & MGP & JKD

MGP = JKD & (! MGP | PHB) | ! MGP & ! PHB & SYS

miRNA165 = SYS | miRNA165

PHB = ! miRNA165 & ! CYA

Auxin = Auxin & ! PHB

IAA5 = ! Auxin

WOX5 = PYI5 & MGP & WYC

CLE = CLE & ! JKD & ! IAA5

ACR = CLE

*SYS = SHR & SCR

*CYA = CLE & ACR

*PYI5 = ! PHB & ! IAA5

*WYC = WOX5 | ! CYA

**Model 6**

SHR = SHR | PHB & ! CYA

SCR = (SYS | JKD) & ! CYA

JKD = ! PHB & MGP | SYS & (! PHB | MGP) | JKD & SYS & ! PHB

MGP = JKD & SYS & ! MGP | (JKD | SYS) & ! Auxin

miRNA165 = SYS | miRNA165

PHB = ! miRNA165 & SHR & (! Auxin | ! CYA)

Auxin = Auxin & ! PHB

IAA5 = ! ( Auxin & ! WOX5 )

WOX5 = (PYI5 | CYA) & SHR & SCR | PYI5 & CYA & SHR

CLE = (CLE | ! SHR) & ! IAA5

ACR = CLE

*SYS = SHR & SCR

*CYA = CLE & ACR

*PYI5 = ! PHB & ! IAA5

**Model 7**

SHR = SYW | ( PHB & ! CYA )

SCR = (SYS | JKD) & ! CYA

JKD = (! PHB | ! JKD) & MGP | SYS & ! PHB

MGP = JYS & ( ! MGP | WOX5 )

miRNA165 = SYS | miRNA165

PHB = ! miRNA165 & ! CYA

Auxin = Auxin & ! PHB

IAA5 = ! Auxin

WOX5 = PYI5 & ! CYA & ! WOX5

CLE = CLE & ! JKD & IYS

ACR = CLE & ! WOX5

*SYS = SHR & SCR

*CYA = CLE & ACR

*PYI5 = ! PHB & ! IAA5

*JYS = JKD | SYS

*IYS = ! IAA5 & ! SHR

*SYW = SHR | WOX5

**Model 8**

SHR = PHB | SHR & ! CLE

SCR = JKD | SYS & PHB

JKD = JKD & SYS & (! PHB | MGP) | ((JKD | SYS) | ! PHB) & MGP

MGP = (JKD & ! SYS) & WOX5 | MGP & (WOX5 | JKD)

miRNA165 = SYS | miRNA165

PHB = ( ! miRNA165 | WOX5 ) & ! CYA

Auxin = Auxin

IAA5 = ! Auxin

WOX5 = PYI5 & ! CYA & JKD

CLE = SYI5 & ( CLE | ! WOX5 )

ACR = CLE

*SYS = SHR & SCR

*CYA = CLE & ACR

*PYI5 = ! PHB & ! IAA5

*SSS = ! SCR & ! SHR

*SYI5 = SSS & ! IAA5

**Model 9**

SHR = SHR & ! CYA

SCR = JKD | SYS & ! IAA5

JKD = ! PHB & ( MGP | SYS ) | JKD & SYS & MGP

MGP = ( JKD | MYS ) & ! WOX5

miRNA165 = ! ( ! SYS & ! miRNA165 & ! JKD )

PHB = ! miRNA165 & ! CYA & SHR

Auxin = ( Auxin | CLE ) & ! PHB

IAA5 = ! Auxin & ! CYA

WOX5 = PYI5 & ! CYA & MGP

CLE =( CLE | ! JKD ) & SYM

ACR = CLE

*SYS = SHR & SCR

*CYA = CLE & ACR

*PYI5 = ! PHB & ! IAA5

*SSS = ! SCR & ! SHR

*SYM = SSS & ! MGP

*MYS = SYS & MGP

**Model 10**

SHR = SHR & ! CYA | (SHR | ! CYA) & PHB

SCR = SYS | JKD

JKD = ! PHB & (MGP | SYS) | JKD & SYS & MGP

MGP = JKD & ! WOX5 (! SYS | ! MGP)

miRNA165 = SYS | miRNA165

PHB = ! miRNA165 & ! CYA

Auxin = Auxin & ! PHB

IAA5 = ! Auxin

WOX5 = PYI5 & ! CYA & MGP | CYA & MGP

CLE = (CLE | ! SHR) & ! IAA5

ACR = CLE

*SYS = SHR & SCR

*CYA = CLE & ACR

*PYI5 = ! PHB & ! IAA5
