# Supplementary material for: Finding Missing Interactions of the Arabidopsis thaliana Root Stem Cell Niche Gene Regulatory Network
Source: Front Plant Sci. 2013 Apr 30;4:110. doi: 10.3389/fpls.2013.00110 (PMC3639504; doi:10.3389/fpls.2013.00110)
Supplement: Supplementary file 3 [file Data_Sheet_3.DOC]

**Predicted interactions**

| **INTERACTION** | **APPEARANCE PERCENTAGE** |
| --- | --- |
| *MGP 🡪 JKD* | 100% |
| *CYA --*| *PHB* | 100% |
| *IAA5* --| *CLE* | 90% |
| *CYA* --| *SHR* | 80% |
| *PHB 🡪 SHR* | 60% |
| SHR --| CLE | 60% |
| *PHB* --| *Auxin* | 50% |
| *MGP 🡪 WOX5* | 50% |
| *CYA 🡪 SCR* | 40% |
| JKD --| CLE | 40% |
| IAA5 --| SHR | 30% |
| *WOX5 🡪 WOX5* | 30% |
| CLE --| SHR | 20% |
| *IAA5 🡪 SHR* | 20% |
| *Auxin --| MGP* | 20% |
| *WOX5 🡪 MGP* | 20% |
| *WOX5 --| MGP* | 20% |
| *SHR 🡪 PHB* | 20% |
| CYA --| IAA5 | 20% |
| *WOX5 🡪 IAA5* | 20% |
| WOX5 --| WOX5 | 20% |
| WOX5 --| CLE | 20% |
| *Auxin 🡪 ACR* | 20% |
| SCR--| CLE | 20% |
| JKD --| SHR | 10% |
| *WOX5 🡪 SHR* | 10% |
| *MGP 🡪 SCR* | 10% |
| *WOX5 🡪 SCR* | 10% |
| *PHB 🡪 SCR* | 10% |
| *CYA 🡪 MGP* | 10% |
| *IAA5 🡪 MGP* | 10% |
| *PHB 🡪 MGP* | 10% |
| JKD --|miRNA165 | 10% |
| *IAA5 --| PHB* | 10% |
| *IAA5 🡪 PHB* | 10% |
| *PHB 🡪 PHB* | 10% |
| Auxin --| PHB | 10% |
| IAA5 --| Auxin | 10% |
| SHR --| Auxin | 10% |
| *SCR 🡪 Auxin* | 10% |
| *IAA5* --| *SCR* | 10% |
| *JKD🡪 miRNA165* | 10% |
| *PHB* --| *SCR* | 10% |
| *CLE 🡪 SCR* | 10% |
| *MGP* --| *CLE* | 10% |
