# Supplementary material for: Finding Missing Interactions of the Arabidopsis thaliana Root Stem Cell Niche Gene Regulatory Network
Source: Front Plant Sci. 2013 Apr 30;4:110. doi: 10.3389/fpls.2013.00110 (PMC3639504; doi:10.3389/fpls.2013.00110)
Supplement: Supplementary file 4 [file Data_Sheet_4.DOC]

Supp Fig 1. Graph of the RSCN GRN with the intermediary nodes included. Intermediary nodes are colored in blue.
